# Complex multiple risk intervention to promote healthy behaviours in people between 45 to 75 years attended in primary health care (EIRA study): study protocol for a hybrid trial

**DOI:** 10.1186/s12889-018-5805-y

**Published:** 2018-07-13

**Authors:** Edurne Zabaleta-del-Olmo, Haizea Pombo, Mariona Pons-Vigués, Marc Casajuana-Closas, Enriqueta Pujol-Ribera, Tomás López-Jiménez, Carmen Cabezas-Peña, Carme Martín-Borràs, Antoni Serrano-Blanco, Maria Rubio-Valera, Joan Llobera, Alfonso Leiva, Clara Vidal, Manuel Campiñez, Remedios Martín-Álvarez, José-Ángel Maderuelo, José-Ignacio Recio, Luis García-Ortiz, Emma Motrico, Juan-Ángel Bellón, Patricia Moreno-Peral, Carlos Martín-Cantera, Ana Clavería, Susana Aldecoa-Landesa, Rosa Magallón-Botaya, Bonaventura Bolíbar

**Affiliations:** 1 Institut Universitarid’Investigació en AtencióPrimària Jordi Gol (IDIAP Jordi Gol), Gran Via Corts Catalanes 587 àtic, 08007 Barcelona, Spain; 2 Gerència Territorial de Barcelona, Institut Català de la Salut, c/Balmes 22, 08007 Barcelona, Spain; 3 Universitat Autònoma de Barcelona, Bellaterra, Cerdanyola del Vallès, Spain; 4 Faculty of Nursing, Universitat de Girona, Carrer d’Emili Grahit, 77, 17003 Girona, Spain; 5 Primary Care Research Unit of Bizkaia, Basque Health Service-Osakidetza, Luis Power Kalea 18, 48014 Bilbao, Spain; 6 Deputy Directorate of Health Promotion, Public Health Agency, Department of Health, Goverment of Catalonia, Roc Boronat, 81-95 (Edifici Salvany), 08005 Barcelona, Spain; 7 Faculty of Psychology, Education and Sport Sciences (FPCEE) Blanquerna, Ramon Llull University, C/Císter 34, 08022 Barcelona, Spain; 8 Faculty of Health Sciences (FCS) Blanquerna, Ramon Llull Univesity, C/Padilla 326-332, 08025 Barcelona, Spain; 9 Parc SanitariSant Joan de Déu, Institut de Recerca Sant Joan de Déu, C/Santa Rosa 39-57, 08950 Esplugues de Llobregat, Spain; 10 Centro de Investigación Biomédica en Red de Epidemiología y Salud Pública (CIBERESP), Madrid, Spain; 11 Gerènciad’AtencióPrimària de Mallorca, Institut de InvestigacióSanitària de les Illes Balears IdISBa, C/Escola Graduada 3, 07002 Palma, Mallorca Spain; 12 Primary Health Centre Vallcarca, Edificio Pedraforca, Av. Vallcarca 169-205, 08023 Barcelona, Spain; 13 Institute of Biomedical Research of Salamanca (IBSAL), Primary Health Care Research Unit, La Alamedilla Health Center, Health Service of Castilla y León (SACyL), Avda. Comuneros 27-31, 37003 Salamanca, Spain; 14 Department of Nursing and Physiotherapy, University of Salamanca, Salamanca, Spain; 15 Department of Biomedical and Diagnostic Sciences, University of Salamanca, Salamanca, Spain; 16 Psychology Department, Universidad Loyola Andalucía, c/Energía Solar 1, Sevilla, Spain; 17 Research Unit, Primary Care District of Málaga-Guadalhorce, c/ Sevilla 23, Málaga, Spain; 18 Institute of Biomedical Research in Málaga (IBIMA), c/ Sevilla 23, Málaga, Spain; 19 El Palo Health Center, Andalusian Health Service (SAS), Av. Salvador Allende 159, 29018 Málaga, Spain; 20 Department of Public Health and Psychiatry, University of Malaga, Campus de Teatinos s/n, 29071 Málaga, Spain; 21 Grupo I-Saúde, Instituto de Investigación Sanitaria Galicia-Sur (IISGS), Xerencia de Xestión Integrada de Vigo, ServizoGalego de Saúde (SERGAS), Universidade de Vigo, Avda Rosalía Castro 21, 36201 Vigo, Spain; 22 Primary Health Centre Beiramar, Xerencia de Xestión Integrada Vigo, Servizo Galego de Saúde (SERGAS), Avda Rosalía Castro 21, 36201 Vigo, Spain; 23 Instituto de Investigación Sanitaria Aragón, Avda. San Juan Bosco 13, 50009 Zaragoza, Spain

**Keywords:** Complex interventions, Cost-effectiveness analysis, Health behaviour, Health promotion, Hybrid trial, Implementation research, Mediterranean diet, Physical activity, Primary health care, Smoking

## Abstract

**Background:**

Health promotion is a key process of current health systems. Primary Health Care (PHC) is the ideal setting for health promotion but multifaceted barriers make its integration difficult in the usual care. The majority of the adult population engages two or more risk behaviours, that is why a multiple intervention might be more effective and efficient. The primary objectives are to evaluate the effectiveness, the cost-effectiveness and an implementation strategy of a complex multiple risk intervention to promote healthy behaviours in people between 45 to 75 years attended in PHC.

**Methods:**

This study is a cluster randomised controlled hybrid type 2 trial with two parallel groups comparing a complex multiple risk behaviour intervention with usual care. It will be carried out in 26 PHC centres in Spain. The study focuses on people between 45 and 75 years who carry out two or more of the following unhealthy behaviours: tobacco use, low adherence to the Mediterranean dietary pattern or insufficient physical activity level. The intervention is based on the Transtheoretical Model and it will be made by physicians and nurses in the routine care of PHC practices according to the conceptual framework of the “5A’s”. It will have a maximum duration of 12 months and it will be carried out to three different levels (individual, group and community). Incremental cost per quality-adjusted life year gained measured by the tariffs of the EuroQol-5D questionnaire will be estimated. The implementation strategy is based on the “Consolidated Framework for Implementation Research”, a set of discrete implementation strategies and an evaluation framework.

**Discussion:**

EIRA study will determine the effectiveness and cost-effectiveness of a complex multiple risk intervention and will provide a better understanding of implementation processes of health promotion interventions in PHC setting. It may contribute to increase knowledge about the individual and structural barriers that affect implementation of these interventions and to quantify the contextual factors that moderate the effectiveness of implementation.

**Trial registration:**

ClinicalTrials.gov, NCT03136211.Retrospectively registered on May 2, 2017.

## Background

Chronic diseases represent a huge personal, social and economic burden and one of the greatest challenges for health systems. They are the leading cause of 68% of the deaths at the global level and approximately 42% of these deaths correspond to people younger than 70 years old. It is estimated that around 80% of cardiovascular diseases and 30% of all cancers could be prevented with the adoption of healthy behaviours: a major portion of these diseases is closely related to smoking, unhealthy diet, sedentary lifestyle, and excessive use of alcohol [1]. Likewise, evidence suggests that diet and physical activity level are important modifiable risk factors for depressive and anxiety disorders [2]. Although social determinants of health play a key role, and there is an important social gradient in the prevalence of risk factors, it is an essential issue to develop effective strategies to cope with them especially in people of low socioeconomic status. Thus, health promotion and prevention is a key process of current health systems with the goals of reducing the risk of disease and disability, contributing to an active and healthy ageing and reducing the need for more expensive health care.

Primary Health Care (PHC) is the most accessible and most commonly used health service which provides an integral and continuous care besides, PHC professionals are the major health-care providers for people with multiple morbidities [3]. That is why PHC is the ideal setting for health promotion and prevention interventions [4, 5]. Furthermore, PHC plays a key part in addressing the social determinants of health, mainly through its role in the community, and contributing, in collaboration with other sectors, to the reduction of social inequalities in health [6]. Moreover, as Barbara Starfield points out, to achieve “more effective, more efficient, safer and more equitable” PHC services, the emphasis should shift from treating diseases to caring for individuals and populations [7]. However, the implementation of health promotion and prevention interventions remains suboptimal mainly as a result of work overload and lack of time or training [8–10]. In addition to all these barriers, the most suitable model to approach behaviour change remains unclear, and there is a lack of theoretical basis of interventions, skills in helping people changing behaviour and knowledge of the local context in which these interventions are undertaken [11–13]. Likewise, there are intrapersonal (beliefs, attitudes, knowledge, skills, self-concept, motivation and resources) and interpersonal (health professionals, family and friends) factors which affect PHC users’ receptiveness to health promotion and prevention interventions [9]. One aspect to keep in mind is that a high percentage of people who visit their PHC professionals tend to have confidence with them and their suggestions have a high impact in PHC users’ everyday life [14].

On the other hand, although the majority of the adult population engages two or more risk behaviours, most of the time the approach is carried out separately when multiple interventions might be more effective and efficient. Nevertheless, most of the studies have only focused on a single behaviour so the knowledge about the effectiveness of a multiple approach is still limited. However, there has been a sustained rise in studies evaluating multiple behaviour interventions since 2002 [15, 16]. These studies show that multiple interventions approach comprising education and skills training are associated with small reductions in risk behaviours [15].

Health promotion and prevention interventions are complex and need an in-depth understanding of the context which contributes to its effectiveness. Regarding this, the methodology proposed by the Medical Research Council offers a unique opportunity [17]. This methodology proposes a development in five sequential phases in which both quantitative and qualitative methods are used, which include: a) definition of the theoretical basis (preclinical phase), b) modelling (phase I), c) exploratory trial (phase II), d) definitive randomised controlled trial (phase III) and e) long-term implementation (phase IV). This methodology promotes the participation of citizens and professionals in research and increases the acceptability and the feasibility of intervention. It is also an ideal tool to achieve the sustainability of interventions and the transfer of research to practice. Research on complex interventions marks a turning point in the conventional way of conducting experimental studies in which the most important thing is finding value and understanding the context of practice rather than trying to control its influence. Hybrid trials represent the ideal design because they allow a joint assessment of clinical effectiveness and implementation, thanks to their dual approach [18]. These trials use theoretical frameworks which ensure a systematic and comprehensive way to understand the determinants of implementation as well as its success and impact [19–21].

In this connection, Spanish primary care prevention and health promotion research network (redIAPP) [22] started in 2012 the EIRA study and carried out the first three phases (preclinical phase, phase I and phase II) [8, 9, 23–25]. Currently, the research team is pursuing the phase III through a hybrid trial which aims to evaluatethe effectiveness, the cost-effectiveness and the implementation of a complex multiple risk behaviour intervention to promote healthy behaviours in people between 45 to 75 years attended in PHC. This article describes the protocol for this trial.

### Study objectives and hypotheses

This hybrid trial has the following primary objectives:

1. To evaluate the effectiveness and cost-effectiveness of a complex multiple risk intervention on reducing tobacco use, enhancing adherence to Mediterranean dietary pattern and increasing physical activitylevel in 12 months to baseline compared with usual care.
2. To assess the effectiveness of an implementation strategy in terms of acceptability, adoption, appropriateness, feasibility, fidelity, implementation cost and penetration.

Furthermore, other secondary objectives related to the effectiveness of the intervention will also be evaluated: its impact on reducing cardiovascular and depression risksas well as depressive and anxiety symptoms and incidence of major depression.

We hypothesise that the proportion of people who show a positive behaviour change with regard to any baseline behaviours will be higher among people who receive the intervention than people who receive usual care. We also hypothesise that the intervention will reduce cardiovascular risk, incidence of major depression, depression risk and depressive and anxiety symptoms.

## Methods

### Design

This study is a randomised controlled hybrid type 2 trial with two parallel groups which aims to test a complex multiple risk behaviour intervention of a maximum duration of 12 months and an implementation strategy simultaneously [18]. The protocol of hybrid trial has been written according to the Standards Protocol Items: Recommendations for Interventional Trials (SPIRIT) [26] and the Standards for Reporting Implementation studies (StaRI) [27]. Cost-effectiveness analysis will be conducted following international recommendations [28].

### Study setting

The study will be carried out in PHC centres of seven of the 17 Spanish Autonomous Communities: Andalusia, Aragon, the Balearic Islands, Basque Country, Castile and León, Catalonia and Galicia. Spanish health system which is based on universal coverage with free access for all citizens, is funded by public sources and depends predominantly on the public sector. PHC is provided by multidisciplinary teams (physicians and nurses, paediatricians, social workers and dentists) who perform activities of health care, health education and prevention. Health promotion and community care are included in the basic PHC services; however, there are multiple barriers, like work overload and lack of time or training, that hinder their implementation [8, 9, 29–31].

### Participants

#### EIRA study has two targets

##### PHC centres

The study comprises 26 PHC centres. The criteria for selecting them are: 1) to have internet access; 2) have the possibility of carrying out community activities; 3) to be not located in areas with a huge cultural and linguistic diversity or in tourist areas and 4) to have a highly committed and active management team. All professionals, healthcare professionals and administrative staff, from PHC centres, will be invited to participate voluntarily. Involved professionals must sign a collaboration commitment to the study.

##### PHC users

The study focuses on people between 45 and 75 years who carry out two or more of the following unhealthy behaviours: tobacco use, low adherence to the Mediterranean dietary pattern or insufficient physical activity level. Participants must provide informed consent before any study procedures occur. In addition, they must be registered with a health professional of the PHC centre. They will be excluded if they have advanced serious illnesses, cognitive impairment, dependence in basic everyday activities, severe mental illness, they are included in a long-term home health care program, they are in treatment for cancer or in end-of-life care, or they do not plan to reside in the area during the time that the intervention lasts.

### Intervention

The intervention is based on the Transtheoretical Model (TTM) [32, 33] and will be made by physicians and nurses in the routine care of PHC practices according to the conceptual framework of the “5A’s”: Assess, Advise, Agree, Assist, and Arrange-follow up [34]. It will consist of a first visit of screening in which PHC professional will assess person level of behaviour and stage of change (“Assess”). Behaviours will be assessed by one question to know tobacco use during the last month, two validate questions about the daily consumption of fruits and vegetables [35] and the Brief Physical Activity Assessment Tool [36, 37]. Stages of change will be assessed on the basis of core constructs of the TTM for each of the target behaviours (see Table 1) [32]. Subsequently, the PHC professional will advise the person (“Advise”), will agree with him/her on a realistic set of goals (“Agree”), will assist to anticipate barriers and will develop a specific action plan (“Assist”), and will arrange follow-up support (“Arrange”).

**Table 1:** Core constructs of the Transtheoretical Model (Prochaska et al. 2008) [32]

Stages of Change	Description
Precontemplation	No intention to take action within the next 6 months
Contemplation	Intends to take action within the next 6 months
Preparation	Intends to take action within the next 30 days and has taken some behavioural steps in this direction
Action	Changed overt behaviour for less than 6 months
Maintenance	Changed overt behaviour for more than 6 months
Termination	No temptation to relapse and 100% confidence

Intervention is based on the results of previous phases (preclinical, phase I and phase II) [23–25, 38–42]. It will have a maximum duration of 12 months and it will be carried out to three different levels (individual, group and community) according to stages of change and unhealthy behaviour (see Table 2). It will focus on all three target behaviours and PHC professional together with the participant will develop priority actions on one or more of these behaviours.

**Table 2:** Description of intervention

	Unhealthy behaviours								
	Tobacco use			Insufficient physical activity			Non-adherence to Mediterranean dietary pattern		
Level of intervention	Individual	Group	Community	Individual	Group	Community	Individual	Group	Community
Stages of change									
Precontemplation	Very brief intervention + SMS			Very brief intervention + SMS			Very brief intervention + SMS		
Contemplation				Brief Intervention + App + SMS	Health education workshops	Social prescribing			
Preparation	Brief Intervention + SMS		Social prescribing				Brief Intervention + App + SMS	Health education workshops	Social prescribing
Action				Very brief intervention + SMS					
Maintenance	Very brief intervention + SMS						Very brief intervention + SMS		
Termination									

*SMS* Short Message Service

Intervention at the individual level has an average intensity between 2 and 3 visits; if necessary, professionals have the freedom to make a greater number of visits. Depending on the stages of change it includes: a) a “very brief intervention” to increase awareness of the need for behaviour change or to support the change and help with relapse prevention; b) a “brief intervention” to make an agreed plan for behaviour change. Health professionals will apply their motivational interviewing skills after following a 20-h online training, an in-person group feedback session and a coded acting patient session [11, 13, 43]. The intervention plan includes the participation in a health education workshop and social prescribing. In addition, the intervention has the support of information and communication technologies, such as a web page addressed to the participant (http://proyectoeira.rediapp.es), the sending of personalised text messages, the use of a mobile app [44] or the recommendation of other gadgets (pedometers, smartwatches, etc).

Group intervention is carried out through two health education workshops focused on healthy diet and physical activity. These workshops are planned to be developed some weeks after initiating the individual intervention and will be conducted by PHC professionals at the health centre. They will take 90–120 min and their purpose is to reinforce the recommendations provided in the individual intervention and to provide people with guidelines that facilitate the practice of physical activity and the adoption of a healthy diet, for example through physical exercise sessions, cooking workshops or preparing seasonal menus.

Community intervention focuses mainly on the social prescribing [45] of resources and activities that are carried out in the community where the participant person resides. Previously every PHC team will identify the community health assets [46] and will choose the most appropriate according to unhealthy behaviours detected, accessibility and the possibility of referral of participants. These interventions will include, for instance, cooking courses, healthy eating workshops, healthy walks, local walking events, line dances, green physical activity programs, etc).

### Usual care

PHC professionals of the control group (usual care) integrate into their practice the recommendations of the Program of Preventive Activities and Health Promotion [47]. This program incorporates preventive protocols that include lifestyle recommendations and a set of preventive activities for a specific age, sex and risk patient groups. Preventive activities are based on systematic screening and brief advice for the prevention of cardiovascular and mental diseases and cancer as well as vaccine recommendations.

### Implementation strategy

The implementation strategy is based on:

- The “Consolidated Framework for Implementation Research” (CFIR) [19] which identifies five constructs: 1) intervention characteristics (intervention source, evidence strength and quality, relative advantage, adaptability, trialability, complexity, design quality and packaging; and cost); 2) outer setting (patient needs and resources, cosmopolitanism, peer pressure, and external policy and incentives); 3) inner setting (structural characteristics, networks and communications, culture, implementation climate and readiness for implementation); 4) characteristics of individuals (knowledge and beliefs about the intervention, self-efficacy, individual state of change, individual identification with the organisation, and other personal attributes); and 5) the implementation process itself.
- A set of discrete implementation strategies [20, 48] which includes: plan strategies (gather information, adapt and pilot material and processes, build buy-in, initiate leadership and develop relationships); educate strategies (develop materials, educate, educate through peers, inform and influence stakeholders); finance strategies (modify incentives and facilitate financial support); restructure strategies (revise professional roles and create community and group interventions committees) and quality management strategies (develop and organise implementation monitoring systems, conduct continuous assessment and feedback, establish a system of reminders, obtain and use patient opinion, centralise technical assistance focused on implementation issues).
- An evaluation framework [21] to determine the effectiveness of implementation through seven implementation outcomes: acceptability, adoption, appropriateness, feasibility, fidelity, implementation cost and penetration.

This implementation strategy will be carried out in three stages, pre-implementation, implementation and post-implementation, further described in Table 3.

**Table 3:** Description of implementation strategies

Stage	Key element	Description
Pre-implementation	Barriers and facilitators	During this stage, the scientific literature will be reviewed. Likewise, the researchers will assess local needs, resources, barriers and facilitators to develop specific implementation strategies. Perspectives of clinicians on the internal resources will be measured by the “Survey of Organizational Attributes for Primary Care”.
	Support materials	All the support material for the intervention will be drawn up.
	Management and quality control systems	Mechanisms for the effective communication and the case report form will be defined and piloted. A checklist (on-line database) will be developed and piloted to monitor the progress of implementation in each PHC centre.
	Facilitation and leadership	The facilitator (member of the research team) and the leader (member of the primary care team) of the implementation will be designated.
	Commitment of the stakeholders	Formal compromises will be made with the managers (at the macro, meso and micro levels) and with the professionals of the centres involved and community partners.
	Training	Training activities will be carried out in which training in motivational interview will have a central role
	Collaborative modelling	Local sessions to adapt and tailor the intervention to the specific context trough shares decisions making.
Implementation	Collaborative learning	The facilitator and the leader of implementation will monitor the implementation processes, identify opportunities for improvement and optimise implementation.
	Commitment of main stakeholders	Audit and feedback techniques will be used towards the main stakeholders in order to keep the agreed compromise and the motivation.
	Training	Health professionals will receive continuous training in motivational interview.
Post-implementation	Management and quality control systems	The evaluation of implementation will be carried out through qualitative and quantitative methodologies

### Outcomes

This study distinguishes between three different but interrelated types of outcomes: i) effectiveness, ii) cost-effectiveness and iii) implementation outcomes.

#### i) Effectiveness outcomes

The effectiveness of the complex multiple risk behaviour intervention in comparison with the usual care at maximum 12 months post-intervention will be measured by:

- *Primary outcome measures*
  Positive change in baseline eating behaviour: adherence to the Mediterranean dietary pattern in low adherence people. For the evaluation, the 14-item Questionnaire of Mediterranean diet adherence (PREDIMED study) will be used [49]. The positive change has been defined as obtaining eight or fewer points at the study entry and nine or more at the end of the study in this questionnaire.
  Positive change in baseline physical activity behaviour: sufficient physical activity level in insufficiently active people. For the evaluation, the International Physical Activity Questionnaire will be used [50]. The positive change has been defined as having a low physical activity level at the study entry and a moderate or high physical activity levelat the end of the study.
  Positive change in baseline smoking behaviour: self-reported continuous abstinence [51]. For the evaluation, the interview will be used and optionally the cooximetry. The positive change has been defined as smoking at the study entry and not smoking at the end of the study. We will measure the punctual and continuous abstinence at these two times.
- *Secondary outcome measures*
  Beginning or making a behaviour change. The proportion of people who are in the stages of action, maintenance or termination according to the TTM at the study entry and at 12 months.
  Change from baseline on sedentary behaviour. It will be measured by the sitting items from the International Physical Activity Questionnaire.
  Change from baseline on diet quality. Diet Quality Index-International will be used to determine diet quality [52].
  Change from baseline on health-related quality of life. It will be measured by the EuroQol-5D questionnaire [53].
  Reduction of the cardiovascular risk. The proportion of people with low/moderate and high/very high baseline cardiovascular risk who have reduced it. Cardiovascular risk will be calculated using REGICOR [54, 55] and SCORE [56] function charts.
  Change from baseline on body mass index. Body mass index is defined as the body weight divided by the square of the body height and is expressed in units of kg/m^2^.
  Change from baseline on waist circumference. The waist circumference will be measured at a level midway between the lowest rib and the iliac crest. It will be expressed in units of cm.
  Change from baseline on blood pressure. It will be measured in the routine clinical practice by validated electronic monitors and it will be expressed in units of mmHg.
  Change from baseline on lipid profile. The lipid profile will include: low-density lipoprotein, high-density lipoprotein, triglycerides and total cholesterol. They will be expressed in units of mg/dl.
  Change from baseline on arterial stiffness. Arterial stiffness will be assessed by the “Cardio-Ankle Vascular Index”. It will be measured by the Vascular Screening System VaSera VS-1500 N or VaSera VS-2000.
  Change from baseline on theankle-brachial index. It will be measured by the Vascular Screening System VaSera VS-1500 N or VaSera VS-2000.
  Change from baseline on the “REgicor and Artper Score fOr aNkle brachial index (REASON)” [57].
  Change from baseline on the perceived functional social support. The questionnaire Duke-UNC-11 will be used to determine the perceived functional social support [58–60].
  Reduction of the incidence of major depression will be evaluated by CIDI interview [61].
  Reduction of the risk of depression in participant non-depressed at baseline. Risk of depression will be calculated using the algorithm PredictD [62].
  Reduction of depression symptoms. The Patient Health Questionnaire-9 will be used to determine the prevalence and the severity of depression symptoms [63].
  Reduction of anxiety symptoms. The General Anxiety Disorder-7 questionnaire will be used to determine the prevalence and the severity of anxiety symptoms [64].
  Change from baseline on unhealthy behaviours of professionals of the PHC intervention centres.

#### ii) Cost-effectiveness outcomes

An economic evaluation will be conducted from the perspective of the society and the Health Service comparing the EIRA intervention vs. the usual care group at 12-months post-intervention.

- *Outcome measure*
  Incremental cost per quality-adjusted life years (QALYs) gained will be calculated. QALYs will be measured using the Spanish tariffs of the EuroQol-5D questionnaire [53]. The following costs will be taken into account: hospital care (emergency visits and stays), secondary care (visits to specialists), primary care (visits to physician and nurse), social care services (visits to social worker), outpatient diagnostic tests, medication use, group sessions attended, community resources used and loss of productivity (days off work). This information will refer to the last 12 months prior to study entry and to the subsequent 12 months. Use of healthcare resources and lost productivity will be assessed through the patients’ clinical history and also the Client Service Receipt Inventory [65]. Information on the use of medicines (active substance, dose, and units supplied) will also be collected. The unit costs of public healthcare services will be obtained from the Official Bulletin of the Government. Costs of privately funded services will be obtained from published tariffs. The mean price per milligram of active substance will be calculated using the prices of the generic versions of all the presentations as reported in the Spanish Vademecum. Productivity losses will be calculated based using information on the minimum and average daily wage in Spain [66].

#### iii) Implementation outcomes

- Early appropriateness and acceptability. It will be evaluated in professionals and participants by means of a survey prior to the start of the intervention.
- Final appropriateness and acceptability. It will be evaluated in professionals and participants by means of a survey. What is more, discussion groups will be held at the end of the intervention with the professionals and participants.
- Adoption. The proportion of professionals who express their willingness to participate in the study between of total of potential professionals prior to the start of the intervention.
- Feasibility. On the basis of the calculation of participation, recruitment and retention rate at 12 months post-intervention.
- Fidelity of the motivational interview model. The quality of the motivational interview delivered will be assessed by coding video recordings of an acting patient session with the “motivational interviewing assessment scale” [67] before and after the training course provided.
- Fidelity of the planned intervention. The degree of compliance of the activities recorded in the case report form (CRF) will be analysed.
- Fidelity of the implementation. The degree of compliance of the implementation strategies.
- Cost of time invested in training and organisational meetings to carry out the intervention.
- Penetration. The proportion of professionals who have integrated the intervention into their usual clinical practice after completing theintervention.

### Sample size

The sample size was calculated on the basis of data from the literature and some results of phase II. We expect a difference in the percentage of people who show a positive change in one or more of the three behaviours between the two groups of at least 8%. Assuming 30% patient loss to follow-up, alpha risk of 5%, beta risk of 20%, and an intracluster correlation of 0.01 [68], we consider that it is necessary to study a minimum of 140 participants for each PHC centre, a total of 3640 people (1820 for each of the two groups, 13 PHC centre per group). PASS software was used to compute the sample size [PASS 14 Power Analysis and Sample Size Software (2016). NCSS, LLC. Kaysville, Utah, USA, ncss.com/software/pass]. Sampling was done in order to fulfil an established sex and age quota which is proportional to the last general population census.

### Recruitment

Several interactive and passive recruitment strategies will be considered to increase the feasibility of achieving the target sample size [69]. Participants will be recruited for hybrid trial at PHC centres through five methods: 1) at the time of visit as part of usual care; 2) self-administered questionnaires delivered in the waiting room or in the admission desk; 3) a part-time training recruiter; 4) advertising by posters in the PHC centres and 5) phone calls to selected patients from review of electronic health records. Method of recruitment should be registered for each participant enrolled. Recruitment will carry out during six months. The facilitator and the leader of the study (see Table 3) will have a crucial role to monitor recruitment. PHC professionals and participants will not receive any financial incentives for enrolment; however, there will be the possibility to set managerial goals related to recruitment.

### Assignment of intervention

The allocation schedule for random assignment of intervention to PHC centres will be computer generated at a central location (IDIAP Jordi Gol, Barcelona, Spain). For each of the seven Spanish Autonomous Communities, we will randomly allocate half of the PHC centres to the intervention group and the other half to the control group. In total, 13 PHC centres will be allocated to the intervention group and 13 other to the control group. The allocation will not be concealed at PHC centres and no blinding will be done.

### Data collection and management

Data will be collected at PHC centre, professional and participant levels. Data collection methods and procedures are summarised in Table 4.

**Table 4:** Schedule of Data Collection Methods and Procedures

			Pre-implementation/Pre-study consent/screening	During implementation			Post-implementation
Activity/Assessment	Data collection	Responsible		Study baseline	Intervention visits	Follow-up 12-months	
PHC centre level							
Characteristics of assigned people	Form report	Research team each autonomous community	x				
Organisational structure	Form report	Research team each autonomous community	x				
Survey of Organizational Attributes for Primary Care	Self-administered questionnaire	Research team each autonomous community	x				x
Professional level							
Age, sex, academic education and experience in PHC	Self-administered questionnaire	Research team each autonomous community	x				
Daily consumption of fruits and vegetables/Level of physical activity/Smoking behaviour	Self-administered questionnaire	Research team each autonomous community	x				x
Appropriateness and acceptability	Self-administered questionnaire	Research team each autonomous community	x				x
Determinants of implementation (CFIR constructs)	Focus group	Research team each autonomous community					x
Participant level							
Informed consent	Paper document	PHC professionals	x				
Age and sex	CRF	PHC professionals	x				
Screening unhealthy behaviours	CRF	PHC professionals	x				
Inclusion/Exclusion criteria	CRF	PHC professionals	x				
Adherence to the Mediterranean dietary pattern	CRF (14-item Questionnaire of Mediterranean diet adherence	External unit of local trained personnel		x		x	
Quality of diet	CRF (Diet Quality Index-International)	External unit of local trained personnel		x		x	
Physical activity behaviour	CRF (International Physical Questionnaire)	External unit of local trained personnel		x		x	
Smoking behaviour	CRF (interview and optional cooximetry)	External unit of local trained personnel		x		x	
Stage of change	CRF	PHC professionals (intervention group)		x		x	
		External unit of local trained personnel (control group)		x		x	
Health-related quality of life	CRF (EuroQol-5D questionnaire)	External unit of local trained personnel		x		x	
Number in the last 12 months of: hospital care (emergency visits and stays), secondary care (visits to specialists), primary care (visits to physician and nurse), social care services (visits to social worker), outpatient diagnostic tests, medication use, group sessions attended, community resources used and loss of productivity (days off work).	CRF Computerised pharmacy records Electronic health record	External unit of local trained personnel		x		x	
Body mass index, waist circumference, blood pressure and lipid profile	CRF	External unit of local trained personnel		x		x	
Cardiovascular risk	CRF (REGICOR and SCORE function charts)	External unit of local trained personnel		x		x	
Arterial stiffness	CRF (measured by the Vascular Screening System VaSera VS-1500 N or VaSera VS-2000)	External unit of local trained personnel		x		x	
Ankle-brachial index	CRF (measured by the Vascular Screening System VaSera VS-1500 N or VaSera VS-2000)	External unit of local trained personnel		x		x	
Functional social support	CRF (questionnaire Duke-UNC-11)	External unit of local trained personnel		x		x	
Risk of depression	CRF (algorithm PredictD)	External unit of local trained personnel		x		x	
Anxiety	CRF (General Anxiety Disorder-7 questionnaire)	External unit of local trained personnel		x		x	
Depression	CRF (Patient Health Questionnaire-9 and the Composite International Diagnostic Interview	External unit of local trained personnel		x		x	
Appropriateness and acceptability of intervention	Self-administered questionnaire	PHC professionals intervention group			x	x	
Determinants of implementation (CFIR constructs)	Focus group	Research team each autonomous community					x

*CFIR* Consolidated Framework for Implementation Research, *CRF* Case Report Form, *PHC* Primary Health Care

At PHC centre level, we will collect information related to: assigned population (total number; average age; distribution by age group and sex; percentage of immigrants; deprivation index; prevalence of tobacco use; level of physical activity and number of people allocated to home care), organisational structure (population coverage; average attendance; number of physicians, nurses and social workers; accreditation as training health centre; participation in undergraduate and postgraduate training; average visits per day; population per health professional; average time per visit and participation research experience). We will also measure the PHC centres’ internal resources for change by the “Survey of Organizational Attributes for Primary Care” [70].

At the professional level, personal data will be collected (age, sex, academic education and experience in PHC). PHC professionals will have to complete a self-administered questionnaire. This questionnaire collects information about professionals’ behaviours with reference to tobacco use, daily consumption of fruit and vegetables and level of physical activity. In addition only in health professionals of intervention centres, it includes nine items to measure the perception of appropriateness and acceptability of the intervention; these items are based in questionnaires which measure similar constructs [71].

Participant-level data are described in Table 4. Recruitment, first screening for entrance to the study and intervention visits will be done by health professionals. Data collection at participant level will be supported by an external unit of local trained personnel who will carry out two visits (baseline and at the 12 months) in each PHC centre because they are not considered as usual care tasks. Data will be collected by means of an online CRF specially designed for the study. This CRF will be pilot tested in pre-implementation stage (see Table 3). The management of the study is based on a central coordination unit, a regional coordination unit and a PHC coordination unit using different periodic communication methods (meetings, teleconferences, e-mails, etc.).

### Analysis

#### Effectiveness of intervention

All analyses will be carried out in accordance with the intention-to-treat principle. Depending on the characteristics of the variables, it will be assessed if it is appropriate to make multiple imputationin the variables with missing data or to make a conservative approach to data loss. Multiple imputation will be the preferred technique for dealing with missing data, and the conservative approach will be used when the data indicate that it is the most appropriate. Multiple imputation by chaining equations procedure will be used to obtain at least 20 imputed datasets and Rubin’s rules will be used to combine them [72]. Sensitivity analyses will be performed for the primary and secondary outcomes to assess the impact of the multiple imputation or the conservative approach to data loss.

Descriptive and bivariate analyses will be calculated for all variables of interest. We will use cluster specific methods because PHC centres will be randomised. In order to analyse the effect of the intervention on each outcome measure, we will use hierarchical linear or logistic regressions models for clustered data. We will also analyse the variables associated with the change in the adherence to the Mediterranean diet, physical activity level and smoking cessation adjusting for possible confounding variables. Final models will be chosen coherently with the study objectives and the nature of the variables (potential confounders, significant and clinically relevant variables). The interactions and collinearity of the models will be evaluated. The size of the effect (Cohen’s d) will also be calculatedand the number needed to treat will also be calculated..

The level of significance of the models will be set at 5%. Stata/SE v.15 or higher (StataCorp, LP, TX) and R version 3.4.4 or higher will be used for statistical analysis.

#### Cost-effectiveness of intervention

The incremental cost-utility ratio will be estimated. It will be obtained by calculating the relationship between the cost of the intervention and the usual care, and its consequences expressed in QALYs.

The cost-utility analysis will refer to the incremental analysis as a result of the quotient between the cost difference of the intervention with respect to usual care, and the change in the results of the difference of cost of the intervention over the difference of cost of the usual care (differences in QALYs). Generalised linear models will be used to model costs and QALYs. Due to the biased distribution of costs, the gamma distribution is usually the most suitable to analyse cost data [73]. The models to estimate differences in costs and QALY will be adjusted for the differences between the two groups in terms of the study covariates. Confidence intervals will be estimated using bootstrap techniques [74] which have the advantage of not having to accept parametric assumptions and whose estimations of standard errors are currently considered very useful and robust in the context of clinical trials [75].

Cost-effectiveness acceptability curves will be obtained based on the theory of net incremental benefit and different hypothetical values of the maximum availability to pay for health gains. These curves represent the probability that an intervention will be more cost-effective than other alternatives and summarise the uncertainty in cost-effectiveness analysis.

Simple sensitivity analyses will be performed. One or more parameters of the evaluation that generate uncertainty will vary in order to see how they affect the robustness of the results. We will conduct a complete case analysis, analysis changing the unit cost of services, andan analysis using the average wage instead of the minimum wage. If the results are sensitive to their variations, simultaneous sensitivity analyses will be performed adjusting for all the sensitive parameters by multiple regression methods.

#### Effectivenessof implementation strategy

The assessment of the implementation strategy will be carried out through qualitative and quantitative methodologies. We will evaluate it in terms of:

- Implementation outcomes (stated earlier).
- Determinants.Characteristics of PHC centres and professionals will be analysed as determinants of implementation. Furthermore, three focus groups (one of the health professionals and two of participants) will be conducted in each PHC centre in the post-implementation stage. Sampling will be theoretical (discursive plurality). Sessions will be transcribed in an anonymous way. A thematic content analysis will be done and data will be coded in accordance with CFIR constructs [76]. Moreover, these results will be analysed quantitatively. CFIR constructs will be scored following standard criteria that will reflect the influence of the construct on the implementation (positive or negative) and its magnitude (between 1 and 2) [77].
- *Level of development*. The level of development of each of the implementation strategies will be determined from an online database. Updating of this database will be carried out by the implementation facilitator. Likewise, we will develop logistic regression models in which the dependent variable will be the effectiveness of the intervention, considered as a positive change in any of the three behaviours studied. The independent variables will be the quantitative measures of the results of the implementation and the degree of implementation of the different strategies. The purpose of these analyses is to model the relationship between the implementation variables and those of effectiveness. On the other hand, the influence and the magnitude of the determinants of the implementation and the results will be established through multivariate models.

## Discussion

Implementation research experts point out that the future studies should be addressed to the sustainable integration of interventions into health care delivery systems [78]. EIRA study is in line with these recommendations as it seeks to achieve changes to promote health through PHC professionals (individual and organisational changes). These changes will reduce the research-practise gap and provide populations health benefits.

To achieve a behaviour change is more feasible when combining individual, group and community interventions [79]. That is why the EIRA study has considered health promotion interventions at these three levels. The study has a person-centred approach that seeks to improve his/her self-efficacy to adopt and sustain healthy behaviours. Because of that, motivational interviewing and information technologies will be used. In addition to focusing on the person, the study has a community-centred focus which aims to increase intersectoral linkages and participatory forums at the local level [30].

However, EIRA study will carry out in real-world PHC setting with a broad range of significant implementation challenges. Implementation of health promotion interventions in PHC is not an easy task, especially after the overall economic crisis, the increasing workload of health professionals and the multiple existing barriers. The previous phases of study enabled to identify some of these barriers and introduced changes in the design of the intervention to improve its feasibility such as reduction of the screening for entrance into the study, incorporation of training on motivational interview and increase in the practical training, extension of the period of follow up, etc. Therefore, the use of the methodology proposed by Medical Research Council has facilitated the design of this study, though some added difficulties have featured. This new approach in research entails an active role of the different stakeholders to create a feasible intervention. This is a big change in the research culture in the present situation of Spanish PHC. Managers, professionals, and researchers are more familiar with the classical methods (clinical trials, for instance) where different factors have to be under control, but not with the uncertainties and variability that implies the adaptation of complex interventions to different PHC centres, and the crucial participation of different stakeholders.

EIRA study will determine the effectiveness and cost-effectiveness of a complex multiple risk intervention and will provide a better understanding of implementation processes of health promotion interventions in this PHC setting. It may contribute to increase knowledge about the individual and structural barriers that affect implementation of these interventions and to quantify the contextual factors that moderate the effectiveness of implementation. This study, therefore, not only entails the evaluation of a complex intervention in PHC but also a change in the evaluation culture of the Spanish research in PHC. The redIAPP, the research network in charge of this study, is introducing this important research perspective in its present scenario.

## Abbreviations

CFIR: Consolidated Framework for Implementation Research
CRF: Case Report Form
PHC: Primary Health Care
QALY: Quality-adjusted life year
SMS: Short Message Service
TTM: Transtheoretical Model
