# Correction to: Complex multiple risk intervention to promote healthy behaviours in people between 45 to 75 years attended in primary health care (EIRA study): study protocol for a hybrid trial

**DOI:** 10.1186/s12889-018-5905-8

**Published:** 2018-08-13

**Authors:** Edurne Zabaleta-del-Olmo, Haizea Pombo, Mariona Pons-Vigués, Marc Casajuana-Closas, Enriqueta Pujol-Ribera, Tomás López-Jiménez, Carmen Cabezas-Peña, Carme Martín-Borràs, Antoni Serrano-Blanco, Maria Rubio-Valera, Joan Llobera, Alfonso Leiva, Caterina Vicens, Clara Vidal, Manuel Campiñez, Remedios Martín-Álvarez, José-Ángel Maderuelo, José-Ignacio Recio, Luis García-Ortiz, Emma Motrico, Juan-Ángel Bellón, Patricia Moreno-Peral, Carlos Martín-Cantera, Ana Clavería, Susana Aldecoa-Landesa, Rosa Magallón-Botaya, Bonaventura Bolíbar

**Affiliations:** 1 Institut Universitarid’Investigació en AtencióPrimària Jordi Gol (IDIAP Jordi Gol), Gran Via Corts Catalanes 587 àtic, 08007 Barcelona, Spain; 2 Gerència Territorial de Barcelona, Institut Català de la Salut, c/Balmes 22, 08007 Barcelona, Spain; 3 Universitat Autònoma de Barcelona, Bellaterra, Cerdanyola del Vallès, Spain; 4 Faculty of Nursing, Universitat de Girona, Carrer d’Emili Grahit, 77, 17003 Girona, Spain; 5 Primary Care Research Unit of Bizkaia, Basque Health Service-Osakidetza, Luis Power Kalea 18, 48014 Bilbao, Spain; 6 Deputy Directorate of Health Promotion, Public Health Agency, Department of Health, Goverment of Catalonia, Roc Boronat, 81-95 (Edifici Salvany), 08005 Barcelona, Spain; 7 Faculty of Psychology, Education and Sport Sciences (FPCEE) Blanquerna, Ramon Llull University, C/Císter 34, 08022 Barcelona, Spain; 8 Faculty of Health Sciences (FCS) Blanquerna, Ramon Llull Univesity, C/ Padilla 326-332, 08025 Barcelona, Spain; 9 Parc SanitariSant Joan de Déu, Institut de Recerca Sant Joan de Déu, C/Santa Rosa 39-57, 08950 Esplugues de Llobregat, Spain; 10 Centro de Investigación Biomédica en Red de Epidemiología y Salud Pública (CIBERESP), Madrid, Spain; 11 Gerènciad’AtencióPrimària de Mallorca, Institut de InvestigacióSanitària de les Illes Balears IdISBa, C/Escola Graduada 3, 07002 Palma, Mallorca, Spain; 12 Primary Health Centre Vallcarca, Edificio Pedraforca, Av. Vallcarca 169-205, 08023 Barcelona, Spain; 13 Institute of Biomedical Research of Salamanca (IBSAL), Primary Health Care Research Unit, La Alamedilla Health Center, Health Service of Castilla y León (SACyL), Avda. Comuneros 27-31, 37003 Salamanca, Spain; 14 Department of Nursing and Physiotherapy, University of Salamanca, Salamanca, Spain; 15 Department of Biomedical and Diagnostic Sciences, University of Salamanca, Salamanca, Spain; 16 Psychology Department, Universidad Loyola Andalucía, c/Energía Solar 1, Sevilla, Spain; 17 Research Unit, Primary Care District of Málaga-Guadalhorce, c/ Sevilla, 23 Málaga, Spain; 18 Institute of Biomedical Research in Málaga (IBIMA), c/ Sevilla, 23 Málaga, Spain; 19 El Palo Health Center, Andalusian Health Service (SAS), Av. Salvador Allende 159, 29018 Málaga, Spain; 20 Department of Public Health and Psychiatry, University of Malaga, Campus de Teatinos s/n, 29071 Málaga, Spain; 21 Grupo I-Saúde, Instituto de Investigación Sanitaria Galicia-Sur (IISGS), Xerencia de Xestión Integrada de Vigo, ServizoGalego de Saúde (SERGAS), Universidade de Vigo, Avda Rosalía Castro 21, 36201 Vigo, Spain; 22 Primary Health Centre Beiramar, Xerencia de Xestión Integrada Vigo, Servizo Galego de Saúde (SERGAS), Avda Rosalía Castro 21, 36201 Vigo, Spain; 23 Instituto de Investigación Sanitaria Aragón, Avda. San Juan Bosco 13, 50009 Zaragoza, Spain

## Correction

It has been highlighted the original article [1] contained a typesetting mistake in the authorship, and that author Caterina Vicens was omitted. This Correction article states the correct authorship. The original article has been updated.
